# Patterns of youth injury: a comparison across the northern territories and other parts of Canada

**DOI:** 10.3402/ijch.v74.27864

**Published:** 2015-08-21

**Authors:** Jessica Byrnes, Nathan King, Penelope Hawe, Paul Peters, William Pickett, Colleen Davison

**Affiliations:** 1 Department of Public Health Sciences, Queen's University, Kingston, ON, Canada; 2 Kingston General Hospital Research Centre, Kingston, ON, Canada; 3 Menzies Centre for Health Policy, University of Sydney, New South Wales, Australia; 4 O'Brien Institute of Public Health, University of Calgary, Calgary, AB, Canada; 5 Department of Sociology, University of New Brunswick, Fredericton, NB, Canada; 6 Department of Economics, University of New Brunswick, Fredericton, NB, Canada; 7 CIHR Team in Child and Youth Injury Prevention, Vancouver, BC, Canada

**Keywords:** adolescent, injury, epidemiology, northern health, population health, alcohol policy, Indigenous health, Aboriginal populations

## Abstract

**Background:**

Injury is the leading cause of death for young people in Canada. For those living in the northern territories (Yukon, Nunavut, and the Northwest Territories), injury represents an even greater problem, with higher rates of injury for people of all ages in northern areas compared with the rest of Canada; however, no such comparative studies have focussed specifically on non-fatal injury in youth.

**Objectives:**

To profile and examine injuries and their potential causes among youth in the northern territories as compared with other parts of Canada.

**Design:**

Cross-sectional data from the 2009/2010 Health Behaviour in School-aged Children survey (youth aged 11–15 years) were examined for the Canadian northern territories and the provinces (n=26,078). Individual survey records were linked to community-level data to profile injuries and then study possible determinants via multilevel regression modelling.

**Results:**

The prevalence of injury reported by youth was similar in northern populations and other parts of Canada. There were some minimal differences by injury type: northern youth experienced a greater percentage of neighbourhood (p<0.001) and fighting (p=0.02) injuries; youth in the Canadian provinces had a greater proportion of sport-related injuries (p=0.01). Among northern youth, female sex (RR=0.87, 95% CI 0.81–0.94), average (RR=0.88, 95% CI 0.80–0.97) or above-average affluence (RR=0.84, 95% CI 0.76–0.91), not being drunk in the past 12 months (RR=0.77, 95% CI 0.69–0.85), not riding an all-terrain vehicle (RR=0.81, 95% CI 0.68–0.97) and not having permanent road access (RR=0.89, 95% CI 0.80–0.98) were protective against injury; sport participation increased risk (RR=1.45, 95% CI 1.33–1.59).

**Conclusions:**

Patterns of injury were similar across youth from the North and other parts of Canada. Given previous research, this was unexpected. When implementing injury prevention initiatives, individual and community-level risk factors are essential to understand; however, specific positive safety assets that might exist in different community contexts must also be considered.

Injury is the leading cause of death for young people in Canada (1, 2). For those living in the northern territories (Yukon, Nunavut, and the Northwest Territories), injury represents an even greater problem. The three territories combined have the highest overall unintentional injury mortality in the country (3). Injury occurrence in Canadian youth has been profiled (4, 5); however, the injury experience of young people living in the northern territories has rarely been examined. The North presents a unique context for life in Canada. Northern communities tend to be smaller, more remote, and have populations with a large proportion of individuals identifying as First Nation, Inuit or Metis (6). There are challenges associated with food insecurity, health services and the provision of affordable housing (7–9), all possible risk factors for injury (10–12). Being male (5), an adolescent (1) and having low socio-economic status are all associated with increased injury risks (13, 14). Although there are many behavioural risk factors associated with injury, excessive alcohol consumption and the use of motorized vehicles are two notable concerns in youth populations throughout Canada (15–18).

Previous studies have determined that the injury experiences of residents of the northern territories differ from those of Canadians as a whole. In addition to higher rates of hospitalizations and mortality, northerners experience a greater proportion of injuries due to self-inflicted harm, off-road vehicle use, being a pedestrian, fighting, assaults and maltreatment compared with southern regions of Canada (19). Few previous studies have focussed their efforts on individual and community exposures and injury experiences specific to northern youth populations, and very few have focussed on the positive assets that might exist in communities for injury prevention. The purpose of this study is to examine injury occurrence among northern youth and to compare injury patterns with those of youth from other parts of Canada. We profile both individual and community-level risk or protective factors for injury. This analysis provides novel evidence to support the development and targeting of specific injury prevention efforts.

## Methods

### Description of data set and study population

To examine injury occurrence, data were taken for secondary analysis from the 2009/2010 (Cycle 6) Health Behaviour in School-aged Children (HBSC) study in Canada. The HBSC survey provides information on youth health behaviours and outcomes, and in 2009–2010, 26,078 youth aged 11–15 years participated (Table I). Of these, 3,942 students attended one of 80 schools located in the three northern territories, and were classified as “northern youth” for this study. The experiences of these students were compared with those from participating provinces. The data set provides information on demographic characteristics, risk behaviours and information for evaluation of injury experiences, including information on injury frequency, location, activity and severity. Community demographic characteristics such as population size and population demographic were determined from the census subdivision (CSD) level data in the 2006 census (Statistics Canada, 2006). Community road access was derived from geographic data and Google maps data (Google Maps and Earth Imagery, 2014).

**Table I:** Overview of data sources for variables examined

Data source	Source information	Variables examined
2009–2010 Health Behaviours in School-aged Children survey	Public Health Agency of Canada and Health Canada. Access facilitated through the Social Program Evaluation Group, Queen's University.	Individual demographics and behaviours • Sex • Grade • Ethnicity • Level of affluence • Impaired driving as passenger or driver in past 30 days • Ride ATVs or other motorized vehicles • Wore a helmet when riding motorized vehicles in the last 12 months • Organized sport participation Outcomes/injury characteristics • Overall injury • Injury severity • Injury frequency • Location of injury • Activity involved in injury
2006 Census data (community level)	2006 Census, Statistics Canada, Census subdivision level data. Data on 1 km school buffers compiled by Dr. Ian Janssen and Andrei Rosu, Queen's University.	Community characteristics • Community population size • Aboriginal composition, by percentage • Dwellings requiring major repair, by percentage
GIS/Google maps	Map data by Google Maps and Google Earth Imagery (2014). Community permanent road access information compiled by Jessica Byrnes.	Community characteristics • Permanent road access
2008 Alcohol Policy community data	Policy database developed by the Population Health Intervention Research Centre, University of Calgary (2005).	Community characteristics • Community alcohol policy (prohibited, restricted or open)

### Outcome variables

The main outcome for this study was a student's experience with injury. Study participants were asked how many times they had been injured and treated by a doctor or nurse during the past 12 months. Response options were “I was not injured in the past 12 months,” or a range from “1” to “4 times or more.” For the most serious injury reported, participants were asked about injury location (“Where were you when this one most serious injury happened?”); activity at time of injury (“What were you doing when this one most serious injury happened”); and injury severity (“did your injury required medical treatment such as the placement of a cast, stitches, surgery or staying in a hospital overnight?” and/or “missed at least one full day from school or other usual activities”). Injuries were deemed severe if they required any of the mentioned treatments or resulted in one or more days of missed activities (5).

### Exposure variables

The HBSC survey asks students to identify their sex and school grade. Relative family affluence was determined by the question: “How well off do you think your family is?” (5 options: “Very well off” through “Not at all well off”). Ethnicity was reported using a number of standard categories including Métis, Inuit or First Nation (20). Students of mixed or multi-ethnic backgrounds could choose up to three options for ethnicity.

Impaired driving behaviours were evaluated using the question: “During the past 30 days, how many times did you drive (or ride in) a car or other vehicle while under the influence, or being driven by someone under the influence of alcohol, using marijuana or other illegal drugs?” [“Never,” “1–3 times” (sometimes) and “4 or more times” (often)]. Off-road vehicle use was determined by “During the past 12 months, how often did you wear a helmet when riding other vehicles (e.g. snowmobile, ATV, dirt bike)?”; participants were categorized as “Yes/riders” if they selected “Never,” “Sometimes,” “Most of the time” or “Always,” and “No” if they selected “I did not ride other vehicles.” Helmet use was inferred directly from this question. Participants were asked, “On how many occasions (if any) have you done the following things? In the past 12 months: been drunk, smoked cigarettes. Responses included “Never,” 1–2, 3–5, 6–9, 10–19, 20–39 and 40 or more times; for study purposes, these were categorized into “Never,” “Sometimes” (1–5) and “Often” (6 or more times). Finally, sports participation was determined by “Do you participate in a sports club or team?” (yes/no).

Population size was categorized as small (<500), medium (500–3,000) or large (>3,000). Communities were classified into groups ranging 0–20%, 21–60%, and >60% of the community with Aboriginal ethnicity. The percentage of dwellings requiring repair in a community was categorized into three groups: <5%, 5–10% and >10%. These cut-points represented approximate tertiles in the distribution of each variable in the northern sample of communities and were determined for the neighbourhood within a 1 km buffer around each school where the survey was administered. Permanent road access, defined as any permanent, year-round access road linking the school community to other communities, was determined using GIS/Google maps (Google Maps and Earth Imagery, 2014). Communities with only seasonal roads or year-round non-road access were not classified under the designation “permanent road access.”

Since the 1970s, local option provisions in provincial and territorial Liquor Control Acts allow communities to decide, through plebiscite, their preferred approach to community alcohol control (21). By hand searching the regulatory database we were able to classify each community as “open/wet” (no community regulations beyond what is found in the Territory's Liquor Act), “restricted/damp” (restrictions to limit the quantity of alcohol that may be imported, consumed, purchased or transported), or “prohibited/dry” (complete ban on the sale, purchase, transport or consumption of alcohol) in 2008.

### Statistical analysis

All analyses were conducted in SAS 9.4 (SAS Institute, Cary, NC). Youth from the northern territories (Yukon, Nunavut, and the Northwest Territories) and other parts of Canada (all provinces except New Brunswick and Prince Edward Island where data were not available) were first described by their individual and home community characteristics. For the children in the Canadian provinces, standardized population weights were applied to ensure representativeness at the provincial level. This was not required for the northern children as a near census was conducted. The Rao-Scott chi-square test, which adjusts for clustering at the school level, was used to assess whether differences between the two regions were statistically significant (p<0.05). Next, the injury experiences of children from the two regions were profiled. Rate ratios, and corresponding 95% confidence intervals, were calculated using the SAS procedure PROC GENMOD.

The final analysis of injury risk factors focussed exclusively on northern children. A multilevel, multivariable, log binomial regression model was used to examine the association between individual and community characteristics, and “any injury.” A priori, the decision was made to keep grade, sex and level of affluence in the final model because of the previously identified strong relationships with injury (1, 5, 14). A backwards elimination approach was used to assess all others.

### Ethics

Ethics approval was granted by the Queen's University General Research Ethics Board, including specific approval for the use of the northern data with student ethnicity. All data were de-identified. Consent was obtained by active, passive or other community-based approaches as per local protocol. The HBSC 2009–10 study was reviewed and approved by the Aurora Research Institute (Northwest Territories), the Nunavut Research Institute and the Heritage Branch of the Yukon Territory Department of Tourism and Culture. The survey is administered in partnership with territorial and provincial government representatives from health and education in each jurisdiction through the Joint Consortium for School Health.

## Results

### Individual and home community context

Table II describes the populations of youth from both the northern territories and the provinces of Canada. Both populations had similar sex and grade distributions; about half of each sample identified as male, and 60% of children from both locations fit into the grade 6–8 category. Over half (53.2%) of northern children reported Aboriginal ethnicity, compared with 6.1% of children residing in other areas of Canada. Self-reported levels of affluence were marginally different between the two populations, with a slightly higher percentage of northern youth falling into the average or not well-off category. Impaired driving, or driving in a vehicle with an impaired driver, in the past 30 days was reported “sometimes” by 8.8 and 5.9% of northern and provincial youth, and “often” by 5.9% of northern youth and 4.2% of provincial youth. All-terrain vehicle (ATV) and motorized vehicle use differed considerably between the two populations; 76.2% of northern youth reported use in the past year, compared with only 52.1% of young people from other parts of Canada. Youth from both populations had similar drinking habits, with about 75.0% reporting having never been drunk in the last 12 months, and approximately 11.0 and 14.0% reporting this behaviour occurring sometimes or often.

**Table II:** Individual characteristics of children from the northern territories and the Canadian provinces

	Northern territories (n=3,942)		Canadian provinces (n=25,991)		
			
	n	%	n	%	P*
Sex					0.39
Male	1,975	50.3	12,772	49.2	
Female	1,949	49.7	13,211	50.9	
Missing	18		8		
Grade					0.91
6–8	2,394	60.7	15,584	60.0	
9–10	1,548	39.3	10,407	40.0	
Ethnicity					<0.001
White	1,514	39.2	19,500	75.8	
Chinese	29	0.8	808	3.1	
South Asian	24	0.6	801	3.1	
Black	32	0.8	771	3.0	
Metis	277	7.2	609	2.4	
Inuit	952	24.6	57	0.2	
First Nation	828	21.4	880	3.5	
Other	207	5.4	2,285	8.9	
Missing	79		278		
Level of affluence					<0.001
Well off	1,851	52.5	13,958	56.9	
Average	1,262	35.8	8,248	33.6	
Not well off	415	11.8	2,330	9.5	
Missing	414		1,456		
Impaired driving in the past 30 days					<0.001
Never	2,905	85.4	21,826	89.9	
Sometimes (1–3 times)	298	8.8	1,433	5.9	
Often (4 or more times)	199	5.9	1,025	4.2	
Missing	540		1,708		
Wore a helmet when riding motorized vehicles (snowmobiles, ATVs etc.) in the last 12 months					<0.001
Never	708	20.8	2,854	11.7	
Sometimes	876	25.7	4,169	17.1	
Always	1,010	29.7	5,671	23.3	
Did not ride other vehicles	812	23.8	11,663	47.9	
Missing	536		1,634		
Been drunk in the last 12 months					0.96
Never	2,693	74.9	18,716	74.9	
Sometimes	400	11.1	2,820	11.3	
Often	501	13.9	3,452	13.8	
Missing	348		1,003		

* P value for Rao-Scott chi-square test.

The home community characteristics varied considerably between the two populations (Table III). Children living in the North more often lived in small communities; over 70% (14.4+57.7%) of northern youth lived in communities of less than 3,000 people, compared with about 35% (5.6+30.9%) of youth from the rest of Canada. Northern communities had a higher percentage of Aboriginal people and had more homes in need of major repairs. Over a quarter (26.2%) of northern children lived in communities without permanent road access, and 51.4% (47+4.2%) lived in communities with prohibitive or restrictive alcohol policies.

**Table III:** Home community characteristics of children from the northern territories and Canadian provinces

	Northern territories (n=3,942)		Canadian provinces (n=25,991)		
			
	n	%	n	%	P*
Community population					<0.001
Small (<500)	566	14.4	1,463	5.6	
Medium (500–3,000)	2,273	57.7	8,042	30.9	
Large (>3,000)	1,103	28.0	16,487	63.4	
% Aboriginal peoples					<0.001
0–20% Aboriginal	1,038	28.9	25,390	98.5	
21–60% Aboriginal	1,454	40.5	309	1.2	
>60% Aboriginal	1,103	30.7	70	0.3	
Missing	347		223		
Permanent road access					
Yes	2,909	73.8	25,991	100.0	
No	1,033	26.2	0	0.0	
Dwellings requiring major repairs					<0.001
<5%	330	8.6	8,067	31.1	
5–10%	1,479	38.6	12,748	49.5	
>10%	2,028	52.9	4,953	19.2	
Alcohol Policy in 2008					
Wet	1,923	48.8	n/a		
Damp	1,852	47.0	n/a		
Dry	167	4.2	n/a		

Note: For “Alcohol Policy in 2008”: “Wet” (open)=no community regulation beyond what is found in the Territory's Liquor Act; “Damp” (restricted or other)=restrictions put in place to limit the quantity of alcohol that may be imported, consumed, purchased or transported; “Dry” (prohibited)=complete ban on the sale, purchase, transport, or consumption of alcohol (21).

* P value for Rao-Scott chi-square test.

### Injuries

Reports of injury experiences were available for 3,682 northern youth and a weighted sample of 25,413 young people from other parts of Canada. No difference in the occurrence of overall injury was observed between the two populations: 41.8% of northern and 41.5% of provincial youth sustained injury within the past year (Table IV). There were also no significant differences found in injury severity or frequency. For both populations, about 17% of children reported a severe injury in the past year, and approximately 21% reported being injured two or more times in the past year.

**Table IV:** Injury experiences of children in the northern territories and the Canadian provinces

	Northern territories		Canadian provinces		
			
	n	%	n	%	Ratio (95% CI)
Overall injury – total	3,682		25,413		
injured	1,539	41.8	10,557	41.5	1.01 (0.95–1.07)
Serious injury – total	3,605		25,183		
Yes	645	17.9	4,385	17.4	1.03 (0.94–1.13)
Frequency of injury – total	3,682		25,413		
0	2,143	58.2	14,856	58.5	1.00 (0.95–1.04)
1	715	19.4	5,250	20.7	0.94 (0.87–1.02)
2+	824	22.4	5,307	20.9	1.07 (0.98–1.17)
Location of injury	3,567		24,907		
Neighbourhood	168	4.7	710	2.9	1.65 (1.36–2.00)
At home	308	8.6	2,390	9.6	0.90 (0.79–1.02)
At a sports facility/field	423	11.9	3,354	13.5	0.88 (0.76–1.03)
At school	255	7.1	1,951	7.8	0.91 (0.78–1.07)
Some other location	282	7.9	1,691	6.8	1.16 (0.99–1.37)
None	2,131	59.7	14,811	59.5	1.00 (0.96–1.05)
Activity involved – total	3,562		24,959		
Sport	715	20.1	5,589	22.4	0.90 (0.82–0.98)
Fighting	108	3.0	541	2.2	1.40 (1.05–1.86)
Riding/driving vehicle	46	1.3	241	1.0	1.34 (0.95–1.87)
Work	18	0.5	108	0.4	1.17 (0.69–1.98)
Walking/running	122	3.4	837	3.3	1.02 (0.86–1.21)
Other	424	11.9	2,815	11.4	1.06 (0.95–1.18)
None	2,129	59.8	14,827	59.4	1.01 (0.97–1.05)

Note: (1) 95% confidence interval adjusted for clustering within schools. (2) “Serious injury” if reported needing medical treatment such as the placement of a cast, stitches, surgery or staying in a hospital overnight. (3) “Neighbourhood” refers to injuries that were reported to have taken place outside in a residential neighbourhood and not in a home, school or sports facility.

When injury occurrences were profiled by location and activity, the distribution of injuries by location or type differed slightly between populations. Northern youth experienced a slightly higher proportion of injuries located in the neighbourhood (4.7% compared with 2.9%, p<0.001) and sustained in fighting (3.0% compared with 2.2%, p=0.01), whereas youth in the provincial subsample experienced slightly higher proportion of sport-related injuries (22.4% compared with 20.1%, p=0.02). No other significant differences were found by injury location or activity. For both populations, a sports facility or field was the most common location for injuries to occur. Sports were the primary activity leading to injury, accounting for over 20% of injuries in both populations.

### Injuries and northern youth

Several individual characteristics and community exposures were shown to be protective against risk of injury among northern youth, after adjustment for potential confounding factors (Table V). Being female and of at least “average” affluence were protective. Certain individual behaviours, such as not getting drunk (RR=0.77, 95% CI 0.69–0.85) or not riding a motorized vehicle (RR=0.81, 95% CI 0.68–0.97) in the past year, were also protective against injury. Organized sport participation was determined to be a risk factor for injury (RR=1.45, 95% CI 1.33–1.59). There appears to be a trend towards injury protection in restricted and dry communities. Higher rates of injury were reported in restricted and open communities compared with prohibited communities, but differences were not statistically significant. Lack of permanent road access was found to be protective against injury (RR=0.88, 95% CI 0.80–0.98).

**Table V:** Results of multivariable regression examining risk of any injury among northern youth by individual and community exposures

	Any injury					
	
			Bivariate		Adjusted	
				
	Total	% Injured	RR	(95% CI)	RR	(95% CI)
Sex Male Female	1,517 1,602	45.3 38.3	1.00 0.85	– (0.78–0.91)	1.00 0.87	– (0.81–0.94)
Grade 6–8 9–10	1,839 1,280	40.6 43.4	1.00 1.07	– (0.98–1.16)	1.00 1.02	– (0.95–1.10)
Level of affluence Not well off Average Well off	349 1,129 1,641	47.3 41.8 40.5	1.00 0.88 0.86	(0.79–0.98) (0.78–0.94)	1.00 0.88 0.84	– (0.80–0.97) (0.76–0.91)
Been drunk in the last 12 months Often Sometimes Never	432 350 2,337	54.6 49.4 38.2	1.00 0.90 0.70	– (0.80–1.03) (0.65–0.75)	1.00 0.96 0.77	– (0.84–1.10) (0.69–0.85)
Impaired driving in the past 30 days Often Sometimes Never	171 268 2,680	49.7 53.4 40.0	1.00 1.07 0.81	– (0.90–1.28) (0.69–0.94)	1.00 1.05 0.91	– (0.88–1.26) (0.79–1.05)
Wore a helmet driving motorized vehicles (ATVs, snowmobiles etc.) in the last 12 months Never Sometimes Always Did not ride a motorized vehicle	637 802 930 750	39.4 47.4 47.0 31.1	1.00 1.20 1.19 0.79	– (1.05–1.38) (1.04–1.37) (0.66–0.94)	1.00 1.12 1.08 0.81	– (0.98–1.29) (0.93–1.26) (0.68–0.97)
Organized sport participation No Yes	1,282 1,837	32.5 48.2	1.00 1.48	(1.36–1.62)	1.00 1.45	– (1.33–1.59)
Alcohol Policy in 2008 Wet Damp Dry	1,594 1,441 84	42.6 41.3 32.1	1.00 0.97 0.75	(0.88–1.07) (0.68–0.84)	1.00 0.99 0.92	– (0.92–1.06) (0.79–1.08)
Permanent road access Yes No	2,442 677	43.2 36.3	1.00 0.84	(0.76–0.93)	1.00 0.89	– (0.80–0.98)

Note: (1) Effect estimates are adjusted for the other variables in the table. (2) Effect estimates are also adjusted for clustering of children within schools.

## Discussion

This study examined the sociodemographic context and patterns of injury occurrence among Canadian children grades 6–10, with a comparison of injury rates between youth in the northern territories and other parts of Canada. Unexpectedly, both populations had similar overall injury prevalence; however, we did identify some differences between northern and other Canadian youth in terms of patterns of injury; and this confirms previous findings (19). Northern youth experienced higher proportions of neighbourhood and fighting injuries and lower proportions of sport-related injury. The slightly elevated risks for neighbourhood injury may be attributable to where and how youth spend their non-school time. If northern youth spend different amounts of time and engage in different activities in neighbourhoods than other Canadian youth, this could have an influence on their injury risk and experiences in these environments. More study on time and space use patterns among youth would also be useful. Time-use patterns have been shown to be different between urban and rural youth (22) with implications for substance use for example.

The finding that fighting injuries occur 36% relatively more often among northern children is sobering, yet consistent with other findings with respect to violence (19) and violent crime (23). In contrast, we noted that youth living in the northern territories had lower prevalence of sport-related injury. As with other context specific injuries, this may be attributable to exposure, as smaller and remote communities may have fewer opportunities for organized sport and recreational activities (24). Despite differences in proportions of sport-related injury, it is still the most prevalent activity associated with injury in both populations, and is an important area of focus for future injury prevention initiatives.

Factors and characteristics found to be protective against injury included being female; of average or high affluence; not being drunk in the past year; not riding an ATV, snowmobile or dirt bike; and living in a community without permanent road access. Although many of these exposures are known protective factors, off-road motorized vehicle use and community road access warrant comment. Many northern youth use ATVs, snowmobiles or dirt bikes (76.2%); helmet use is variable. Northern populations experience a proportion of injuries associated with the use of these kinds of vehicles that is higher than the Canadian average (19). Although not riding is protective, it may not be reasonable to suggest ATV or snowmobile disuse, as they are a principal mode of transportation in Canada's north (25). Instead, injury prevention initiatives should focus on risk management, for example, education and enforcement of safe use of motorized vehicles, or discouraging recreational ATV or snowmobile use by children (25). Additionally, impaired driving among youth and those using ATVs is an important concern (26) and essential to focus upon. In this study, wearing a helmet was found to increase risk of injury; however, this anomaly may be attributed to the fact that helmet use likely indicates greater frequency of motorized vehicle use and more time or opportunity for risk.

Our finding of equivalent prevalence of injury in northern as compared with other Canadian youth was unexpected given past literature describing the numerous risk factors for injury in rural, remote and northern communities, as well as above average rates of injury and injury mortality previously reported in northern populations (3, 27–30). There might be a number of explanations for our disparate results. First, past studies have focussed more on severe injuries or injury-related fatalities (3, 4) and included adults. Injury patterns may differ when considering injuries of different levels of severity, injury mortality, or among population of varied ages. Second, injury incidence may indeed be higher in northern environments, but we are capturing fewer injuries among northern youth because of health care access issues, as the HBSC measures injuries “treated by a doctor or nurse.” While many northern communities have nurses and nursing stations, fewer have doctors or other health care staff on a permanent basis. If there were differential access to, or use of, health care services for injuries, this would impact our ability to compare the two groups. A third explanation for the unexpected parity in northern and provincial youth injury could relate to possible protective factors that may exist in northern families and communities that may mediate the effects of hazardous exposures if they are indeed greater in northern settings. For example, while populations in rural and remote communities have been identified as being at increased risk of injury and other health disadvantages, dry communities in northern regions have reported overall fewer alcohol-related hospitalizations and lower rates of violent crime than other communities (31, 32). We have found in ongoing analysis with HBSC data that youth in dry communities consume less alcohol on average than their peers in wet or damp communities (paper in preparation). In our study of injury in youth, there appears to be an encouraging trend for communities with alcohol prohibition and restriction to have lower injury rates (see unadjusted and adjusted estimates in Table V). Presence of a community alcohol policy was not statistically significantly associated with risk for injury after adjustment for other factors; however, in analytic assessment of this, we determined that it was the addition of the variable “permanent road access” in the fully adjusted model that accounted for the reduction in the alcohol policy influence. Knowing the northern community context, this is not surprising. While not completely collinear, community policy option does tend to distribute roughly by community remoteness, where fly-in communities represent most of the dry communities (i.e. only one of the dry communities had permanent road access). Not having permanent road access, a proxy measure for remoteness, is associated with social and physical environmental factors that may influence youth injury. These factors include, for example, the number and type of vehicles in a community; existence and condition of community infrastructure like playgrounds or sports fields; and the diversity or overall number of adults available to take on roles of oversight, care or mentorship. These characteristics may represent important factors in the prediction of youth injury. No permanent road access may also reduce injuries associated with riding or driving in vehicles such as cars or trucks. By adjusting for both alcohol policy option and permanent road access, some of the influence of these factors may have been hidden. However, these analyses point towards important potential safety assets at a community level. Future analyses could explore these and additional community factors as unique predictors to further establish potential aetiologic pathways.

Strengths of this study include the size and representative nature of the sample, which permitted subanalyses by specific injury characteristics. The comparison between the northern territories and youth in other parts of Canada gives researchers and policy-makers a frame of reference when making decisions regarding where injury prevention efforts need to be focussed. Another strength of this study lies in our use of advanced multilevel models, accounting for clustering that occurs at the school or community-level, to simultaneously examine associations between individual and community characteristics, and injury. This study was also one of the first to examine non-fatal injury among a population based sample of northern youth; many previous studies focussed only on injury mortality or injuries presenting in a hospital setting.

This study also has limitations. Weaknesses stemming from the HBSC survey include its self-reported nature and the fact that it is cross-sectional and does not allow definitive tests of causation. Some analyses yielded wide confidence intervals; this could be improved by larger sample sizes or a different analytical approach. Northern communities in the HBSC sample were identified as those in any of the three northern territories; we recognize there are other definitions of “northern” where areas of the provinces might also be included. In addition to lacking information on fatal injury outcomes and the physical nature of injuries, the HBSC survey does not differentiate unintentional injury from intentional injury. Intentional injuries are a leading cause of hospitalizations and death throughout Canada (1, 2), particularly within the North (27, 28), and it could be beneficial for future studies to examine intentional injuries uniquely.

Public health efforts centred on injury prevention can benefit from the findings of this research, which encourages acknowledgement of the similarities and differences between northern youth and those living in other parts of Canada. It also encourages emphasis on positive safety assets that might exist within communities. In some northern communities, restrictions on alcohol availability or lack of road access (with its implications for vehicle use) both appear protective for example. Targeting safe sport participation is important. Variations in the distribution of injuries by location and activity, although minimal, reveal areas where prevention efforts could also be focussed. In any youth population, careful consideration of the context in which injuries occur and knowledge of community-specific exposures is essential to create effective and successful injury prevention programmes. Our findings indicate that community and socio-economic characteristics influence youth injury risk. Prevention efforts should focus in these areas in addition to individual behavioural interventions.
